# Proteomics analysis of the gut–brain axis in a gut microbiota-dysbiosis model of depression

**DOI:** 10.1038/s41398-021-01689-w

**Published:** 2021-11-08

**Authors:** Yiyun Liu, Haiyang Wang, Siwen Gui, Benhua Zeng, Juncai Pu, Peng Zheng, Li Zeng, Yuanyuan Luo, You Wu, Chanjuan Zhou, Jinlin Song, Ping Ji, Hong Wei, Peng Xie

**Affiliations:** 1 NHC Key Laboratory of Diagnosis and Treatment on Brain Functional Diseases, The First Affiliated Hospital of Chongqing Medical University, Chongqing, China; 2 Department of Laboratory Animal Science, College of Basic Medical Sciences, Third Military Medical University, Chongqing, China; 3 College of Stomatology, Chongqing Medical University, Chongqing, China

**Keywords:** Molecular neuroscience, Depression

## Abstract

Major depressive disorder (MDD) is a serious mental illness. Increasing evidence from both animal and human studies suggested that the gut microbiota might be involved in the onset of depression via the gut–brain axis. However, the mechanism in depression remains unclear. To explore the protein changes of the gut–brain axis modulated by gut microbiota, germ-free mice were transplanted with gut microbiota from MDD patients to induce depression-like behaviors. Behavioral tests were performed following fecal microbiota transplantation. A quantitative proteomics approach was used to examine changes in protein expression in the prefrontal cortex (PFC), liver, cecum, and serum. Then differential protein analysis and weighted gene coexpression network analysis were used to identify microbiota-related protein modules. Our results suggested that gut microbiota induced the alteration of protein expression levels in multiple tissues of the gut–brain axis in mice with depression-like phenotype, and these changes of the PFC and liver were model specific compared to chronic stress models. Gene ontology enrichment analysis revealed that the protein changes of the gut–brain axis were involved in a variety of biological functions, including metabolic process and inflammatory response, in which energy metabolism is the core change of the protein network. Our data provide clues for future studies in the gut–brain axis on protein level and deepen the understanding of how gut microbiota cause depression-like behaviors.

## Introduction

Major depressive disorder (MDD) is a serious mental illness characterized by low mood, loss of motivation, feelings of despair, and an inability to feel pleasure. MDD is one of the leading causes of disability worldwide [1], and >50% of patients do not remit after first-line antidepressant treatment despite numerous advances in the pharmacological treatment of depression [2]. Many studies have reported that depression is highly correlated with the activity of inflammatory signals [3, 4], alterations in neurotrophic signals [5], deficits in brain reward processing [6, 7], abnormal activity of the hypothalamic–pituitary–adrenal axis [8], changes in DNA methylation [9], and DNA damage [10]. However, these theories do not adequately explain the pathogenesis of depression. Interestingly, there is increasing evidence from both animal and human studies suggesting that the gut microbiota are actively involved in driving depression-like behaviors [11, 12] and provided new potential targets for MDD therapy [13].

Gut microbiota have been reported to regulate brain development, function, and behavior [14–16]. The use of germ-free (GF) mice maintained in a sterile environment allows assessment of how gut microbiota shape brain function and behavior. Bidirectional communication between the microbiota and brain via the gut–brain axis may contribute to the risk of neuropsychiatric diseases through alterations in the gastrointestinal system, central nervous system, autonomic nervous system, and immune systems [14, 17]. Moreover, recent studies have confirmed profound effects of the microbiome on neuropsychiatric diseases such as autism spectrum disorder [18], schizophrenia [19], and Alzheimer’s disease [20] via gut–brain axis.

In our previous clinical study, several altered metabolic byproducts of gut microbiota were found in urine of MDD patients, including hippurate, dimethylamine, and dimethylglycine [21]. We subsequently found that the gut microbial communities of MDD patients were significantly different from those of healthy controls [12], and these alternations were relatively specific compared to that observed in schizophrenia [22]. Moreover, based on a humanized fecal microbiota transplantation (FMT) animal model, we demonstrated that mice recipients of MDD fecal samples displayed depression-like behavior at 2 weeks post-transplantation and showed a significant disturbance of carbohydrate metabolism and amino acid metabolism compared with control mice [12]. We also found that gut microbiota caused molecular changes in multiple tissues, including the hippocampus, liver, cecum, serum, and hypothalamic–pituitary–adrenal axis [12, 23–25]. Mass spectrometry-based proteomics is a powerful approach to precisely quantify thousands of proteins in complex samples and to identify novel differentially expressed (DE) proteins between pathological states and controls [26, 27], which has obvious advantages in discovering new targets and distinguishing psychiatric disorders [28, 29]. Thus, proteomics may be an effective way to reveal the underlying mechanisms of how the gut microbiota impact on hosts at the protein level.

The aim of this study was to capture the key protein alterations involved in the gut–brain axis. To this end, the gut microbiota of MDD patients was transplanted to GF mice. We then performed quantitative proteomics to examine the prefrontal cortex (PFC), liver, cecum, and serum. We hypothesized that gut microbiota would affect the host’s gut–brain axis at the protein level and induce the depression-like behaviors.

## Materials and methods

### Animals

**Fig. 1:**
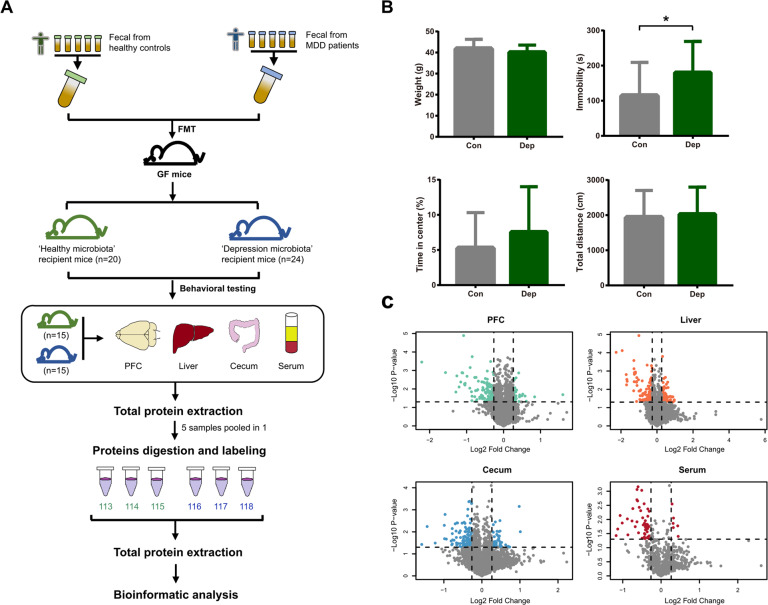
Workflow of this study. **A** Experimental and proteomics workflow of the study. **B** There were no differences in weight, total motion distance, and percentage of time spent in the center of the OFT between “depression microbiota” and “healthy microbiota” recipients. The “depression microbiota” recipients displayed an increased duration of immobility in the FST compared with “healthy microbiota” recipients (*p < 0.05, *t* test). The gray bars indicate the “healthy microbiota” recipients (*n* = 20). The green bars indicate the “depression microbiota” recipients (*n* = 24). **C** Volcano plots of detected protein abundance from the four tissues. Green, orange, blue, and red dots indicate DE proteins in the PFC, liver, cecum, and serum, respectively.

GF Kunming male mice (aged 6–8 weeks, weight 30–40 g) were obtained from the Experimental Animal Research Center of the Third Military Medical University (Chongqing, China). GF mice were kept in flexible film gnotobiotic isolators until the beginning of the behavioral tests. Mice were housed in standard autoclaved polypropylene cages with access to food and water ad libitum under a 12-h dark–light cycle (light on at 07:30) and at a constant temperature (23 ± 1 °C) and relative humidity (55% ± 5%). All animal handling and procedures followed the recommendations of the Guide for the Care and Use of Laboratory Animals and were approved by the Ethics Committee of Chongqing Medical University (Chongqing, China). Experimental and proteomics workflow of this study is shown in Fig. 1A.

### Fecal microbiota transplantation

Written informed consent was obtained from all participants. Participants aged 18–60 years were recruited from the psychiatric center and medical examination center of the First Affiliated Hospital of Chongqing Medical University. Eligible patients were drug-naive with a primary diagnosis of MDD as assessed by the Structured Psychiatric Interview using Diagnostic and Statistical Manual of Mental disorder, Fourth Edition-Text Revision criteria [30] by an experienced psychiatrist. The score of 17-item Hamilton Depression Rating Scale of MDD patients were >18 (mean [SD] score = 26.8 [4.0]). Exclusion criteria included a current diagnosis of physical or other mental disorders and substance abuse. Healthy controls were excluded if they had a history of neurological or other Axis I/II disorders, alcohol abuse or dependence, and illicit drug use. There were no differences in demographic characteristics between two groups. Fecal samples obtained from MDD patients (*n* = 5, 3 females, mean [SD] age = 37.4 [13.0]) and healthy controls (*n* = 5, 2 females, mean [SD] age = 42 [12.9]) were used to colonize the guts of GF mice. The protocol for microbiota transplantation was as previously described [12]. Briefly, fecal samples were collected under anaerobic conditions. We took 0.1 g of stool from each sample, then pooled five samples from the MDD or healthy group, respectively. The pooled 0.5 g of fecal sample for each group was suspended with 7.5 ml of 0.9% sterile phosphate-buffered saline to obtain suspension. GF mice were then randomly colonized with fecal samples from MDD patients or healthy controls in the flexible film gnotobiotic isolator.

### Behavioral testing

Two weeks after the FMT, the open field test (OFT) and the forced swim test (FST) for animals were performed within 2 days. After each individual test session, the apparatus was cleaned with 75% alcohol. All behavioral tests were recorded and analyzed by the EthoVision XT software (Noldus, Wageningen, Netherlands).

For the OFT, all mice were individually tested in an open-field apparatus (45 × 45 × 45 cm). After 1 min of adaptation, all spontaneous activities were recorded for 5 min using a video tracking system [31]. For each animal, the total distance and percentage of time spent in the center quadrants were used to evaluate the anxiety-like behavior. For the FST, after 1 min of adaptation, all mice were individually placed into glass cylinders (30 cm high, 15 cm in diameter) containing 18 cm of water at 23 ± 2 °C for 5 min [32]. All sessions were recorded with a video tracking system. Immobility was defined as the least amount of movement needed to stay afloat. Duration of immobility were used to evaluate the depression-like behavior.

### Sample collection and preparation

After behavioral tests, the whole brain was rapidly removed, and PFC was dissected from the brain. Liver and cecum samples were also rapidly obtained. All tissues were quick frozen in liquid nitrogen and then stored at −80 °C. Serum was immediately separated by centrifugation at 3000 rpm for 20 min at 4 °C and then stored at −80 °C. Samples from 15 FMT-treated mice with depression-like phenotype and 15 control mice were prepared for proteome fractionation. The sample size was calculated by the power analysis with a Cohen’s *d* effect size of 0.8. All tissues were homogenized in SDT buffer (4% SDS, 100 mM Tris-HCl, 1 mM DTT, pH 7.6) [33]. Proteins from five mice per group were pooled as a biological sample, and three biological replicates were obtained for each group. Proteins were digested with trypsin (Promega, Madison, WI, USA) in dissolution buffer overnight at 37 °C. Peptides were purified on C18 Cartridges (Empore™ SPE Cartridges C18, bed I.D. 7 mm, volume 3 ml; Sigma, Steinheim, Germany), concentrated by vacuum centrifugation, and reconstituted in 0.1% (v/v) formic acid.

### iTRAQ labeling and strong cation exchanger (SCX)-based fractionation

The pooled samples were labeled using iTRAQ reagent according to the manufacturer’s instructions (Applied Biosystems, Foster City, CA, USA). We used six tag of each iTRAQ 8-plex reagent ranging from 113 to 118. Four iTRAQ-labeling reagents were used for the 24 pools from the four tissues. iTRAQ-labeled peptides were fractionated by SCX chromatography using the AKTA Purifier system (GE Healthcare, Waukesha, WI, USA). The mixed iTRAQ-labeled samples were dissolved in buffer A (10 mM KH_2_PO_4_ in 25% of ACN, pH 3.0) and were then eluted at a flow rate of 1 ml/min with a gradient of 0–8% buffer B (500 mM KCl, 10 mM KH_2_PO_4_ in 25% of ACN, pH 3.0) for first 22 min, 8–52% buffer B from 23 to 47 min, 52–100% buffer B from 48 to 50 min, 100% buffer B from 51 to 58 min, and buffer B was reset to 0% after 58 min. The elution was monitored by absorbance at 214 nm, and fractions were collected every 1 min. For each experiment, 33 fractions were collected.

### Liquid chromatography tandem mass spectrometry (LC-MS/MS) and data analysis

Each fraction was injected for nano-LC and MS analysis. The peptide mixture were separated on a reverse phase trap column (Thermo Scientific Acclaim PepMap100, 100 μm × 2 cm, nanoViper C18; Thermo Fisher Scientific, Waltham, MA, USA) connected to a C18-reversed phase analytical column (Easy column, 10 cm long, 75 μm inner diameter, 3 μm resin; Thermo Fisher Scientific, Waltham, MA, USA) in buffer A (0.1% formic acid) and then separated with a linear gradient of buffer B (84% acetonitrile and 0.1% formic acid) at a flow rate of 300 nl/min controlled by IntelliFlow technology. LC-MS/MS analysis was performed on a Q Exactive mass spectrometer outfitted with an Easy nLC (Thermo Scientific). The MS detection was survey scan (300–1800 *m*/*z*) with an target automated gain control value set of 3e6 and a maximum inject time of 10 ms. A dynamic exclusion time of 40 s was used. Survey scans were acquired at a resolution of 70,000 at *m*/*z* 200, then the resolution for high-energy collisional dissociation spectra was set to 17,500 at *m*/*z* 200, and isolation width was set to 2 *m*/*z*.

Obtained MS/MS spectra were processed with Proteome Discoverer 1.4 (Thermo Fisher Scientific). The processed data were searched with Mascot version 2.2 (Matrix Science, London, UK). The mouse protein database was downloaded from Uniprot (released November 4, 2016) with 81,798 total entries. Two missed cleavages were allowed in fully and partially tryptic peptides. Carbamidomethyl (C), iTRAQ 8-plex (N-term), and iTRAQ 8-plex (K) were set as fixed modifications, and the oxidation (M) and iTRAQ 8-plex (Y) were set as variable modifications. The peptide mass tolerance was 20 ppm, and fragmentation tolerance was 0.1 Da. All peptide ratios were normalized by the median protein ratio. False discovery rate (FDR) were determined using a concatenated target-decoy database, and the peptides were identified with 1% FDR [34]. Proteins were considered to have differential abundance with a one-sample *t* test *p* value <0.05 and a fold change >1.2.

### Statistical and bioinformatics analysis

All statistical tests were performed with the statistical software SPSS (version 17.0; Chicago, IL, USA). Kolmogorov–Smirnov and Shapiro–Wilk tests were used to assess the normality of the behavioral testing data and weight. Student’s *t* test was performed to compare differences between the two groups. A *p* value <0.05 was considered to indicate statistical significance. To further understand the similarities and differences of the detected proteins between the humanized FMT model and chronic stress models of depression, we compared the PFC data from the chronic social defeat stress (CSDS) model [35] and the liver data from chronic unpredictable mild stress (CUMS) model [36] reported in our previous studies. Moreover, we included our previous olfactory bulb (OB) data of FMT model in the bioinformatics analysis [37].

The protein datasets of different tissues were independently processed by weighted gene coexpression network analysis in the R software (version 3.6.1) [38]. We applied minimum module size to 20 proteins and the minimum height for merging modules at 0.25 to obtain the modules. Gene Ontology (GO) terms and models’ overlap were performed by the Metascape [39]. Enrichment of GOs and DE proteins in modules were determined by Fisher’s exact test corrected with Benjamini–Hochberg (BH) adjusted *p* value <0.05. Protein annotation for functional groups and cellular compartment is obtained from the Ingenuity Pathway Analysis (IPA) software (QIAGEN Bioinformatics). Protein–protein interaction (PPI) networks were constructed by the STRING (version 11.0) and Cytoscape (version 3.7.2).

## Results

### Changes in gut microbiota induced depression-like behaviors

Two weeks after FMT, behavioral tests were performed to evaluate whether gut microbiota affected the function of the brain. There was no significant difference in body weight between the two groups (*t* = 1.34, *p* = 0.19; Fig. 1B). In the OFT, there were no differences in the total distance or percentage of time spent in the center between the “depression microbiota” recipient mice and control mice (*t* = −0.42, *p* = 0.68; *t* = −1.30, *p* = 0.20). In the FST, the immobility time of “depression microbiota” recipient mice was significantly increased compared with controls (*t* = −2.38, *p* = 0.02). These results were consistent with our previous studies [12, 25], suggesting that disturbances of gut microbiota affect behaviors.

### Protein expression profile of gut–brain axis

Large-scale protein analysis was performed on the PFC, liver, cecum, and serum from two groups. We identified 27,445 unique peptides in the PFC with an FDR < 1%, covering 4846 proteins. With the same parameters, a total of 4858 proteins (29,096 unique peptides) were identified in the liver, 4167 proteins (22,036 unique peptides) in the cecum, and 848 proteins (4504 unique peptides) in serum (Tables S1–S4). All identified proteins of four tissues showed a large range of abundance, and the distributions of proteins varied among tissues (Fig. 1C).

**Fig. 2:**
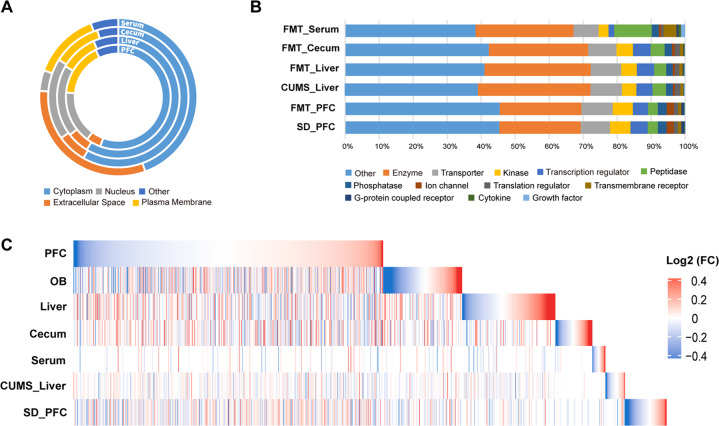
Protein expression profile. **A** Donut plots represent the percentages of cellular compartments of all proteins. **B** Composition of the functional groups annotated by IPA. In the PFC, about 2.1% of proteins involved in ion channel functions, which was approximately twice as high as that in the liver, cecum, and serum. The proportion of G protein-coupled receptors in the PFC was also three times higher than that in the cecum and liver. In the serum, the numbers of growth factors, cytokines, transmembrane receptors, and peptidases were markedly higher than that in the other three tissues. **C** Union heatmaps show fold changes of all proteins for the gut–brain axis of FMT model, the PFC of CSDS model, and the liver of CUMS model.

Analysis of protein location and functional annotation by IPA revealed that protein location of serum was different from the other three tissues (Fig. 2A and Tables S5–S8). All proteins were assigned to 13 functional groups (Fig. 2B). We found that the proportion of various functional proteins detected in the cecum and liver was similar, but it was different between the PFC and serum (Fig. 2B). Further, the union heatmap of protein expression levels showed obvious separations among the four tissues (Fig. 2C).

In order to further reveal the similarities and differences of the detected proteins between the humanized FMT model and chronic stress models of depression, we compared the data of PFC from the humanized FMT model and CSDS model and the data of liver from humanized FMT model and CUMS model. In general, 67.1% of PFC proteins of FMT model overlapped with CSDS model, and 55.8% of liver proteins of FMT model overlapped with CUMS model (Fig. S1A). However, only 1.9 and 1.1% of DE proteins of FMT model overlapped with CSDS and CUMS models, respectively (Fig. S1B). For the same tissue from different models, the protein expression showed divergence, indicating that microbiota-driven protein changes were different from the chronic stress.

### Comparisons of differential protein expression among tissues

We identified 159, 187, 148, and 55 DE proteins in the PFC, liver, cecum, and serum, respectively (Tables S1–S4). Further, the majority of DE proteins were downregulated (Fig. S2A). There were no DE proteins that overlapped in all four tissues (Fig. S2B). Five DE proteins overlapped between the PFC and cecum, and six overlapped between the liver and cecum. For brain tissues, PFC and OB shared ten DE proteins as well. These results suggested that gut microbiota exerted different impact on multiple tissues of the gut–brain axis.

### Coexpression analysis identified microbiota-related protein modules

**Fig. 3:**
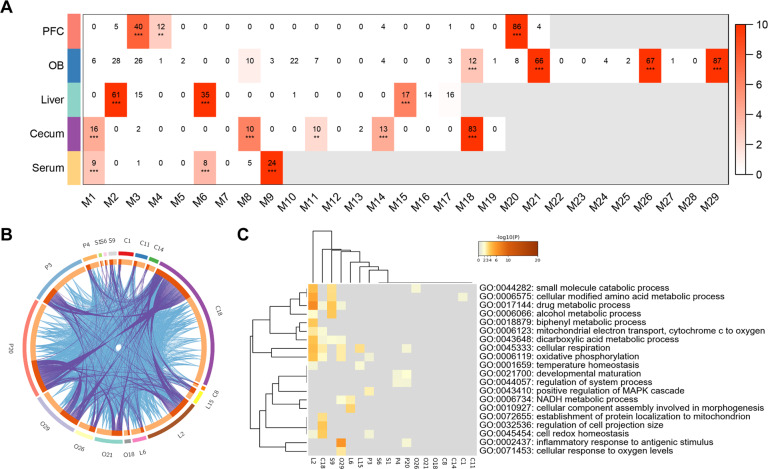
DE proteins and microbiota-related coexpression modules. **A** Matrix summarizes enrichment of DE proteins in coexpression modules for each tissue. The significance of enrichment (−log10 (BH-corrected *p* value), one-sided Fisher’s exact test) is scaled by color intensity. BH-corrected **p < 0.01, ***p < 0.001. **B** Circos plot shows overlapped proteins in modules from PFC, OB, liver, cecum, and serum. The outside arcs of circus plot represent each module, and the inside arcs represent the proteins in each module. Dark orange represents the proteins that appear in multiple module and light orange represents proteins that are unique to that module. Purple links indicate the overlapped proteins among modules, and blue links indicate the functional overlap among modules. **C** GO enrichment analysis for 18 microbiota-related modules. The heatmap cells are colored by the *p* values of representative GO terms.

To assess the proteome-wide changes among tissues of gut–brain axis in a more comprehensive manner, we constructed independent protein coexpression networks based on all detected proteins from five tissues. We identified 21, 29, 17, 19, and 9 coexpression modules in the PFC (P1–P21 modules), OB (O1–O29 modules), liver (L1–L17 modules), cecum (C1–C17 modules), and serum (S1–S9 modules), respectively (Fig. S3), ranging from 22 to 1631 proteins. In total, 18 modules were enriched for DE proteins in the gut–brain axis, which were considered as microbiota-related modules (FDR < 0.05, Fig. 3A). The circus plot of these modules suggested that the microbiota-related modules across the gut–brain axis showed similar protein expression (Fig. 3B).

To examine the biological function of the microbiota-related modules, we conducted GO enrichment analysis (Fig. 3C). These modules were extensively involved in the metabolic process, including small molecule catabolic process (adjusted *p* = 0.002), cellular modified amino acid metabolic process (adjusted *p* = 0.007), drug metabolic process (adjusted *p* < 0.001), and dicarboxylic acid metabolic process (adjusted *p* < 0.001). Modules of PFC and OB were involved in inflammatory response to antigenic stimulus (adjusted *p* < 0.001). Notably, L2, C18, O29, P3, and P20 were all enriched in the cellular respiration (adjusted *p* < 0.001) and oxidative phosphorylation (adjusted *p* < 0.001), which are critical energy metabolism pathways.

**Fig. 4:**
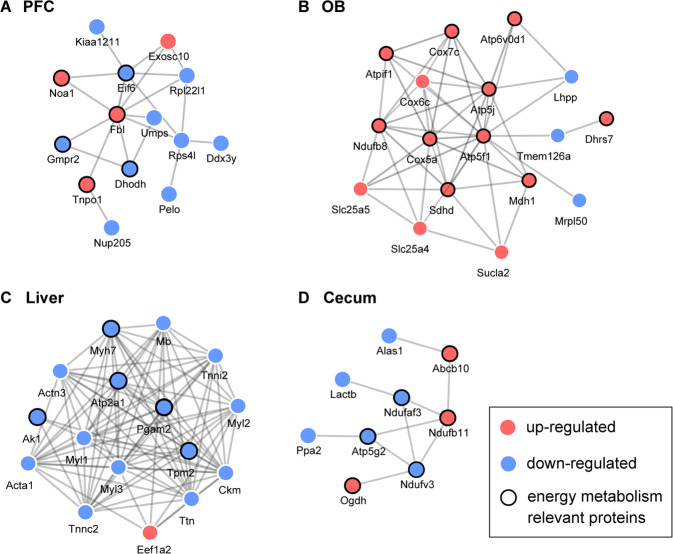
The PPI networks of the gut–brain axis. The PPI network of the **A** PFC, **B** liver, **C** cecum, and **D** serum based on the DE proteins. Blue dots indicate downregulated DE proteins. Red dots indicate upregulated DE proteins.

Further, we constructed the PPI networks based on the DE proteins of the gut–brain axis (Fig. S4) and annotated the protein functions by GO. In the PPI networks of PFC and cecum, proteins involved in the energy metabolism formed separate subnetworks (Fig. 4A, C). In the OB and liver, energy metabolism-relevant proteins showed a tight link and played important roles in the structure of the entire PPI networks (Fig. 4B, D). Together, these results suggested that DE proteins were highly enriched in energy metabolism across tissues of gut–brain axis and might have worked in a synergistic way.

## Discussion

Increasing evidence supports that gut microbiota are important contributors to the balance between mental health and disease [13]. In the present study, a humanized FMT model was used to induce depression-like behavior, and an iTRAQ-based quantitative proteomics approach was used to explore protein expression of the gut–brain axis. Our results supported that gut microbiota induced altered protein expression levels in multiple tissues of the gut–brain axis in mice with depression-like phenotype, and these changes of the PFC and the liver showed to be model specific. In addition, protein changes of the gut–brain axis were involved in a variety of biological functions, including metabolic process and inflammatory response, in which energy metabolism is the core change of protein networks.

As is well known, the gut microbiota can modulate host’s functions in energy uptake, storage, and expenditure. Microbes can increase energy harvest through the short-chain fatty acids produced by fermentation [40]. We found that the gut microbiota not only changed the energy metabolism of cecum but also had profound effect on the PFC, OB, and liver. Moreover, our model is based on the GF mice, and these gut–brain axis protein changes involved in energy metabolism may be a cause of depression-like behaviors, rather than a consequence. This suggests that the effect of gut microbiota could transmit from intestinal tract to brain. These findings are consistent with previous studies which showed that the altered gut microbiota could impact the host’s metabolism [12, 40, 41]. Besides, increasing preclinical studies suggested that the gut microbiota might induce depression-like phenotype via the vagus nerve [42, 43] and the subdiaphragmatic vagotomy blocked behavioral changes in knockout mice treated with an antibiotic cocktail [44, 45].

Metabolomics studies have revealed that depression patients showed disturbance of energy metabolism both in urine and plasma [21, 46]. In a proteomics study, postmortem dorsolateral PFC brain tissue of patients with MDD showed significant differences in energy metabolism [47]. Our previous study found that the liver of the FMT-treated mice with depression-like phenotype also presented energy metabolism change on metabolite level [24]. In a transcriptomic study, depression-like behaviors were accompanied with mitochondrial energy metabolism as well [48]. These studies on multiple molecular types supported that depression was closely related to energy metabolism.

Our results showed that 5-hydroxytryptamine receptor 2A (HTR2A), a member of the serotonin receptor family, was significantly upregulated in the PFC of mice with depression-like phenotype, which is consistent with clinical studies of MDD patients [49–51]. HTR2A is highly associated with depression severity and plays an important role in the serotonin signaling pathway of depression [52]. Most of the body’s 5-HT are produced in the gut and regulates its movement. Gut microbiota were reported to regulate host’s 5-HT and physiology via the colonic enterochromaffin cells [53]. The gut-driven 5-HT alteration could lead to abnormal liver gluconeogenesis and glucose uptake through HTR2B [54]. These data indicated that gut microbiota might alter the expression of neurotransmitters in the brain to influence the behaviors.

Based on the comparison of datasets, we found that over half of detected proteins in the humanized FMT model overlapped with chronic stress models; however, only approximately 1–2% of DE proteins overlapped between these models. The DE proteins of the humanized FMT model differed markedly from the chronic stress models of depression in both PFC and liver. These results suggested that microbiota might have a novel pathogenic mechanism, which differed from common psychological and physical stress. Moreover, previous studies reported that the depression-like mice of CSDS model showed a distinct microbiota composition compared with controls [55]. Further, the depression-like Flinders sensitive line rats, which are used as an experimental animal model of depression, showed lower bacterial richness and altered relative abundance of several bacterial phyla [56]. These studies suggested that the onset of depression often followed with alterations in microbiota composition. Based on the FMT model of depression, our data demonstrate that depressive-like phenotypes can be transmitted from human to mice.

Our study had several limitations. First, we used male GF mice to construct the humanized depression model. Nevertheless, other studies showed that females were 2–3 times more likely to develop MDD and suffered greater functional impairment [57, 58]. Besides, although GF mice showed limitation in development and immunity [59], they are still valuable for exploring whether or not gut microbiota impact a given process [60]. Therefore, the future studies using female GF mice and antibiotics-treated mice can provide a supplement to our results. Moreover, as all recipient mice were kept in flexible film gnotobiotic isolators characterized by a higher interior air pressure to avoid potential contamination of forage and air, we did not perform the sucrose preference test, a measurement for the behavior of anhedonia that is a core symptom of MDD [61]. Also, our behavioral tests were performed out of the isolators, and it is necessary to explore the effect of short-term air exposure on the composition of gut microbiota in subsequent experiments. In addition, only four tissues related to the brain–gut axis were investigated in our study, while changes in microbiota were also reported to affect other tissues, including the heart and pancreas [62, 63].

In conclusion, using a gut microbiota-dysbiosis model of depression, we found that gut microbiota may have an essential role in the development of depression-like behaviors and contribute to changes of the protein expression and functions of the gut–brain axis, especially the energy metabolism. Our data provide clues for future studies on the gut–brain axis at the protein level and deepen the understanding of how gut microbiota cause depression-like behaviors.

## Supplementary information

Supplementary figure legends

Supplementary figure 1

Supplementary figure 2

Supplementary figure 3

Supplementary figure 4

Supplementary Table 1–8
