# Supplementary material for: Proteomics analysis of the gut–brain axis in a gut microbiota-dysbiosis model of depression
Source: Transl Psychiatry. 2021 Nov 8;11:568. doi: 10.1038/s41398-021-01689-w (PMC8572885; doi:10.1038/s41398-021-01689-w)
Supplement: Supplementary file 1 — Supplementary figure legends [file 41398_2021_1689_MOESM1_ESM.docx]

**Supplementary Figure Legends**

**Fig. S1.** (A) Proteins overlapped between chronic social defeat stress (CSDS) model and fecal microbiota transplantation (FMT) model (*left*); proteins overlapped between chronic unpredictable mild stress (CUMS) model and FMT model (*right*). (B) Differential expression (DE) proteins overlapped between CSDS model and FMT model (*left*); differential expression proteins overlapped between CUMS model and FMT model (*right*).

**Fig. S2.** (A) DE proteins in serum, cecum, liver, and PFC. (B) Venn diagram displaying the overlap among tissues.

**Fig. S3.** Clustering dendrogram of proteins with dissimilarity based on topological overlap matrix. One color represents one module.

**Fig. S4.** PPI networks based on the DE proteins of PFC, liver, cecum, and olfactory bulb.
