# Supplementary figures and images for: Proteomics analysis of the gut–brain axis in a gut microbiota-dysbiosis model of depression
Source: Transl Psychiatry. 2021 Nov 8;11:568. doi: 10.1038/s41398-021-01689-w (PMC8572885; doi:10.1038/s41398-021-01689-w)

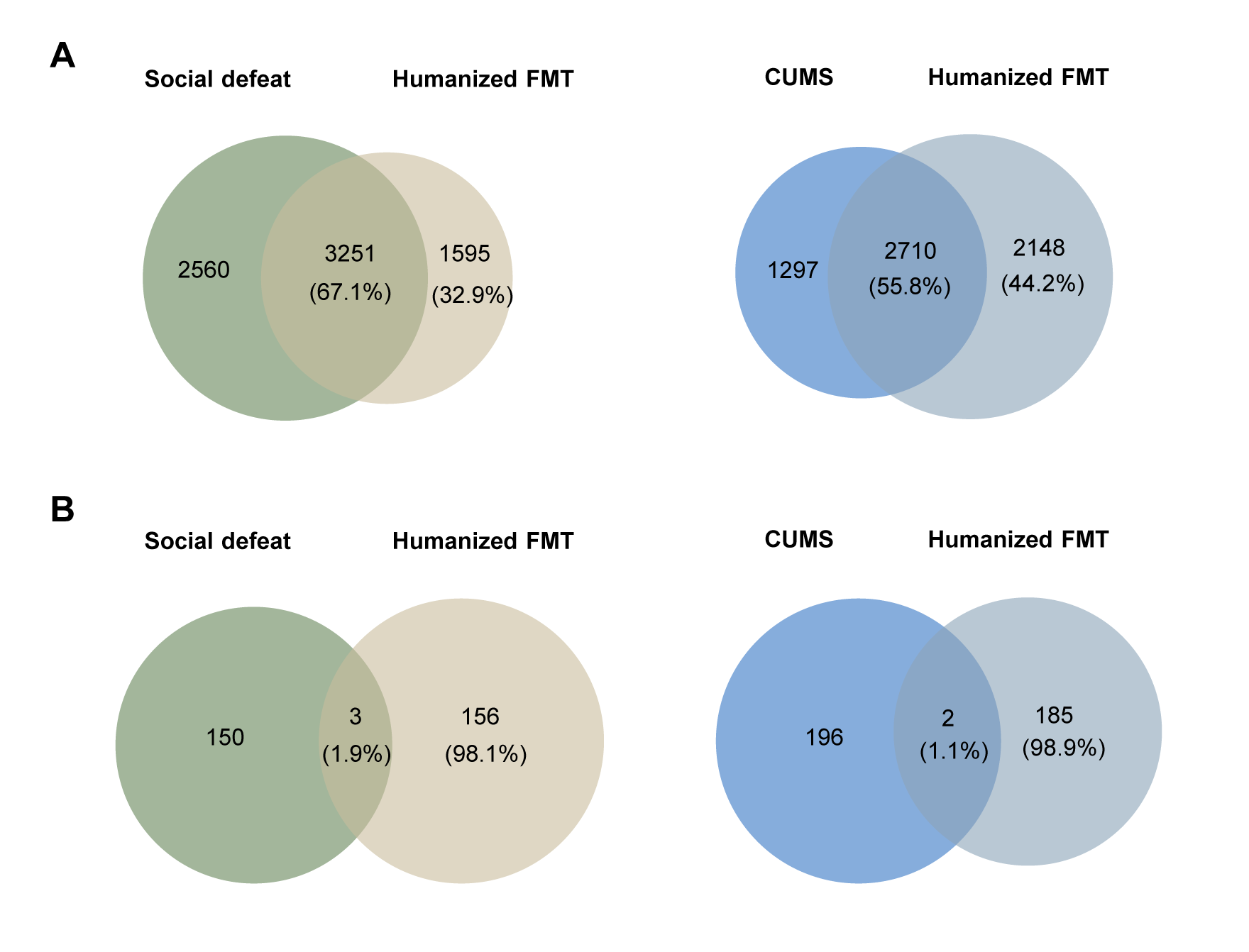

Supplement: Supplementary file 2 — Supplementary figure 1 [file 41398_2021_1689_MOESM2_ESM.tif]

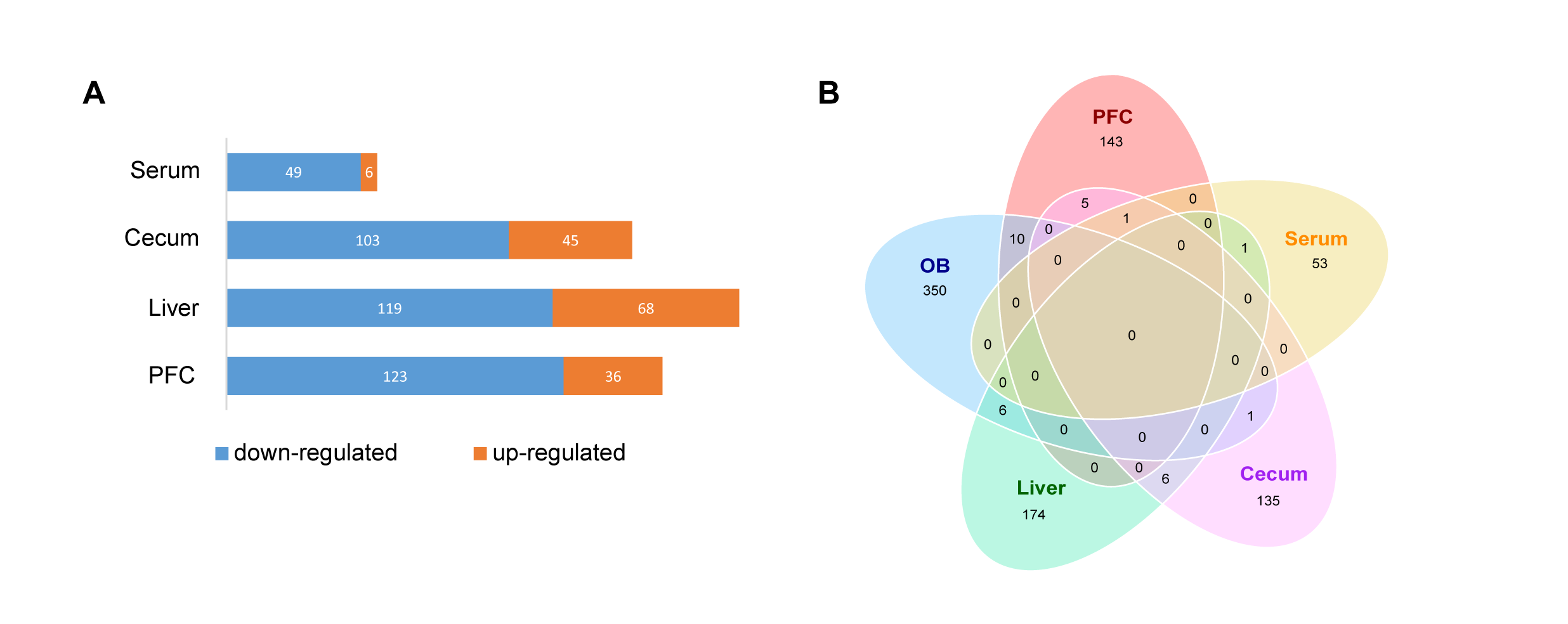

Supplement: Supplementary file 3 — Supplementary figure 2 [file 41398_2021_1689_MOESM3_ESM.tif]

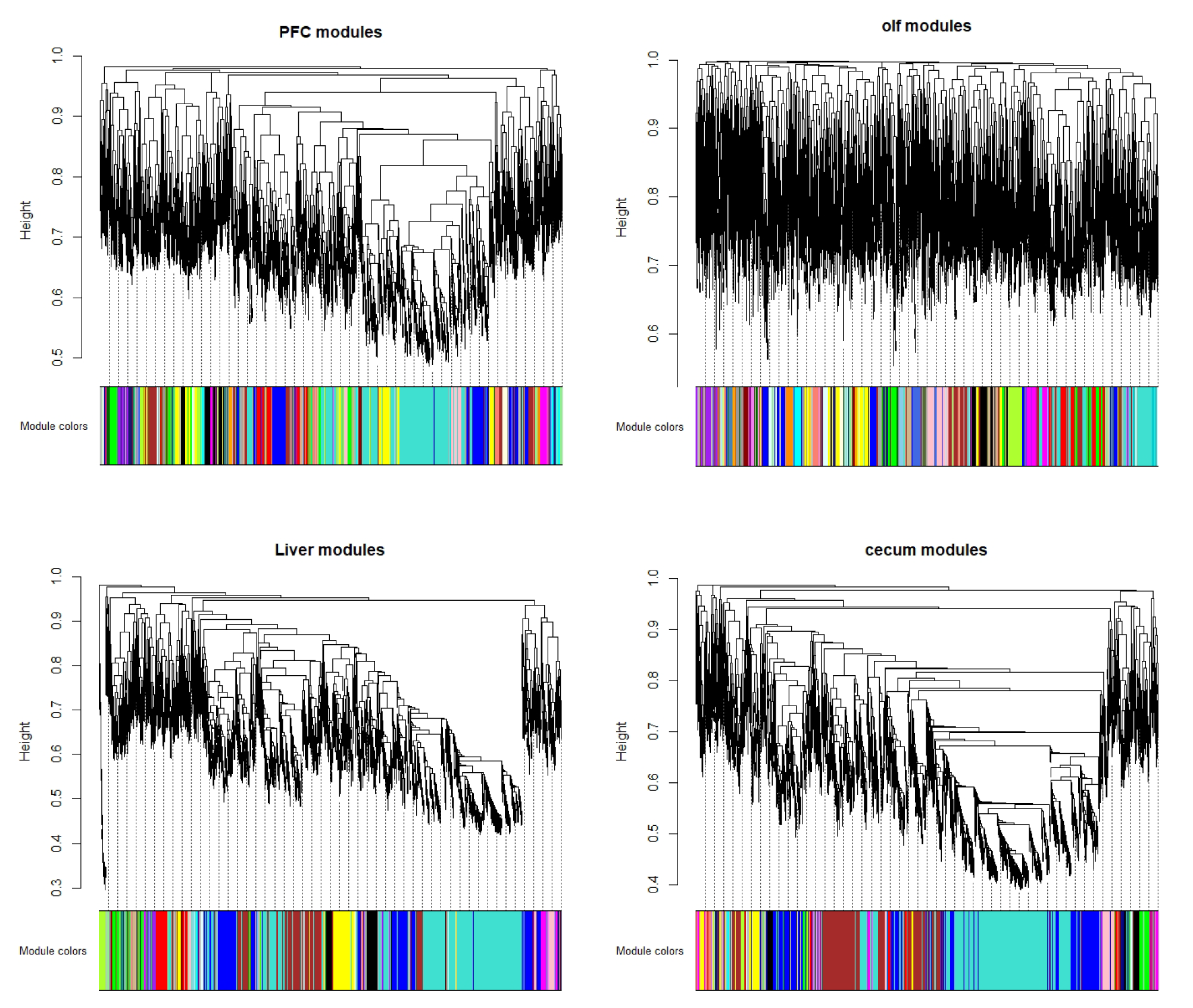

Supplement: Supplementary file 4 — Supplementary figure 3 [file 41398_2021_1689_MOESM4_ESM.tif]

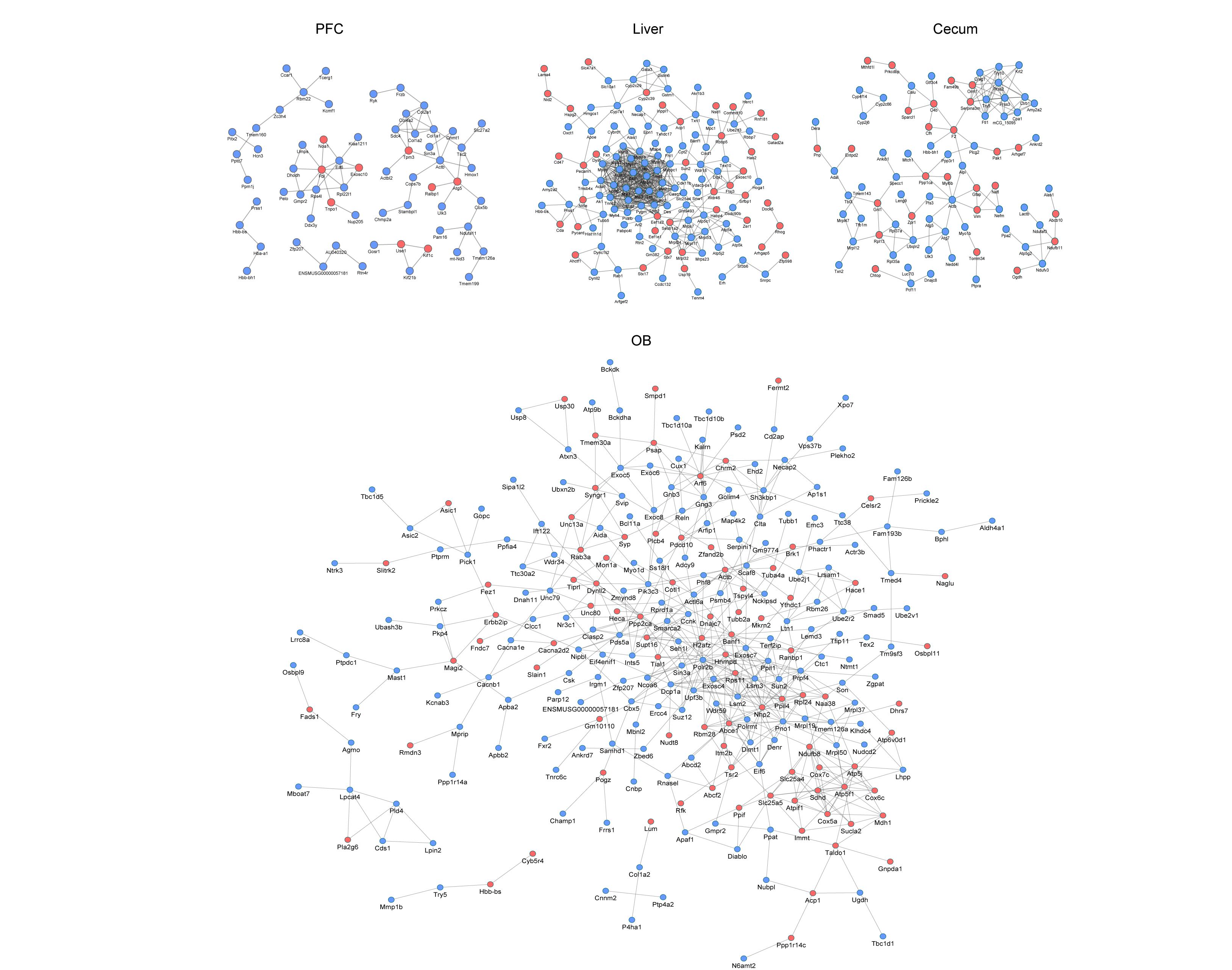

Supplement: Supplementary file 5 — Supplementary figure 4 [file 41398_2021_1689_MOESM5_ESM.tif]
